# The Effect of Monensin vs. Neem, and Moringa Extracts on Nutrient Digestibility, Growth Performance, Methane, and Blood Profile of Merino Lambs

**DOI:** 10.3390/ani13223514

**Published:** 2023-11-14

**Authors:** Danah A. Du Preez, Abiodun Mayowa Akanmu, Festus Adeyemi Adejoro, Abubeker Hassen

**Affiliations:** 1 Department of Animal Science, University of Pretoria, Private Bag X20, Hatfield, Pretoria 0028, South Africa; 2 School of Biosciences, University of Nottingham, Sutton Bonington, Leicestershire LE12 5RD, UK

**Keywords:** feed additives, plant secondary metabolites, rumen fermentation, toxicity

## Abstract

**Simple Summary:**

Modulating the rumen environment can help to improve the efficiency of feed utilization, minimize methane gas production, or improve the overall health of the animals. Leaf extracts of Moringa and Neem plants were tested as potential phytogenic rumen modulators in growing merino lambs while also evaluating the effect on the performance and health of the animals. The doses tested did not have any negative effect on the health and performance of the animals. However, the leaf extracts from Moringa and Neem tended to reduce methane emissions, and thus necessitating testing of higher inclusion levels and standardization of the plant extract using key bioactive compounds associated with methane inhibition.

**Abstract:**

Plant secondary compounds are potential rumen modifiers that can improve nutrient utilization in ruminant animals. This study evaluated the effect of Moringa (*Moringa oleifera*) and Neem (*Azadirachta indica*) leaf extracts on nutrient digestibility, growth performance, and enteric methane production in South African Mutton Merino lambs. Forty 4-month-old ram lambs with a mean body weight of 35 ± 2.2 kg were blocked by weight and from each block, lambs were randomly allocated into one of the following treatments: (i) diet only (fed a total mixed ration TMR-negative control), (ii) Monensin (fed TMR containing Monensin sodium, 15 mg/kg DM), (iii) Moringa (fed TMR, drenched with Moringa extract 50 mg/kg feed DM intake), and (iv) Neem (fed TMR, drenched with Neem extract 50 mg/kg DM intake). Extracts were administered via oral drenching at a concentration determined based on the previous week’s feed intake. There were no differences in dry matter intake, average daily gain, feed conversion efficiency, digestibility, and nitrogen retention across the treatments. However, the extracts tended to reduce methane emitted both in g/head/day (*p* < 0.08) and g/ kg dry matter intake (*p* < 0.07). Extracts did not influence any of the blood metabolites in the ram lambs. Although the benefits of utilizing these medicinal plants as rumen modifiers under prolonged feeding conditions is justified, further evaluation is recommended to test Moringa and Neem leaf extracts at higher inclusion levels. Our research group is currently exploring a variety of phytogenic tools for the identification and standardization of key bioactive compounds linked to methane inhibition, in these leaf extracts.

## 1. Introduction

Ruminant livestock contributes significantly to global methane emission, and several enteric methane mitigation strategies are being explored with the hope of advancing climate-smart livestock production strategies [1,2,3]. Ionophores, probiotic inoculants, defaunating agents, medicinal plants, and their constituent plant secondary metabolites (PSMs) are a few examples of feed additives that have shown varying degrees of enteric methane reduction [4,5]. Aside from the direct impact of these feed additives on methanogenesis, the associated effects on nutrient intake and digestibility, animal performance and health are important factors to consider before they can be recommended for farmers’ adoption [2,6].

Medicinal plants or extracted PSMs are dietary additives with wide biological diversity, with more than 200,000 defined bioactive substances already identified from a wide array of medicinal plants [7]. Furthermore, these PSMs have been reported to show strong antioxidant, hepatoprotective, anthelmintic, antiviral, and growth-promoting properties in human and animal models at varying doses [8,9]. Feed additives such as PSMs could offer a cost-effective approach to reducing animal-derived methane, especially where it has co-benefits such as enhancing feed utilization, animal performance, and health [10]. 

*Moringa oleifera* (Moringa) and *Azadirachta indica* (Neem) are medicinal plants with a wide biological activity and high concentration of PSMs within their foliage, thus making them potential rumen modulating and enteric methane mitigation agents [8,11,12]. These effects are associated with the presence of tannins, saponins, flavonoids, alkaloids, and terpenoids [6,13]. The potential adaptation of rumen microbes to additives after prolonged feeding time limits the application of in vitro data while digesta outflow from the rumen; the impact of additives on animal health can only be evaluated with live animals [6,14,15]. Most of the studies evaluating the efficacy of Neem and Moringa were in vitro procedures, and some evaluated the foliage as the sole substrate. In an in vivo study by Kholif et al. [16], increased feed intake, nutrient digestibility, and higher short-chain fatty acid (SCFA) production were observed when a 20 mL oral dose of Moringa leaf extract was administered to Nubian goats. Similarly, Malik et al. [6] reported a 26% reduction in methane emissions and significant defaunation of the rumen entodinimorphic protozoa population when the sheep diet included 4% Neem leaves. In these studies, methane emissions were estimated from SCFA concentration using an equation [1] and only a slight decrease in methane production was reported [16]. 

There remains a dearth of information on the in vivo methane mitigation potential of Moringa and Neem leaf extract, as well as the potential impact on performance and health. Blood parameters are reliable markers of animal health and can be indicative of potential toxic effects when animals consume feeds containing any antinutritional factor [17]. We hypothesize that oral administration of Neem and Moringa extracts at the dose of 50 mg/kg DM intake to growing lambs will result in reduced enteric methane production without any negative effect on the growth and blood profile of the animals. Therefore, the objective of the study was to evaluate the effect of Neem and Moringa leaf extract on growth performance, nutrient digestibility, methane production, and blood profile of South African Mutton Merino lambs.

## 2. Materials and Methods

Animal management protocols were carried out in accordance with the South African guidelines on the care and use of experimental animals and approved by the Animal Ethics Committee of the University of Pretoria under approval number EC-004-18.

### 2.1. Animal, Experimental Design, and Adaptation 

Dried foliage of Neem and Moringa was milled to pass through a 1 mm sieve and extracted in 100% methanol. The methanolic extract was evaporated in a fume cupboard, freeze-dried and 1 g crude extract was subsequently reconstituted in 1 L of distilled water and stored as a stock solution at 4 °C [14]. Forty 4-month-old SA Mutton Merino lambs were grouped by body weight, and from each group, two lambs were housed in a pen and randomly allocated in a completely randomized design into one of the following dietary treatments: (i) diet only (fed a total mixed ration TMR-negative control), (ii) Monensin (fed TMR containing Monensin, 15 mg/kg DM), (iii) Moringa (fed TMR, drenched with Moringa extract 50 mg/kg feed DM intake), and (iv) Neem (fed TMR, drenched with Neem extract 50 mg/kg DM intake). The lambs were housed in open-sided pens made of concrete floors and metal roofing, and a total of 10 lambs in 5 pens were assigned to each dietary treatment. Each lamb was drenched with the daily dosage in two equal portions at 08h00 and 16h00, using a 20 mL metal drenching gun (NJ Philips, Brisbane, Australia) and coinciding with feeding time when the total mixed ration (Table 1) was offered. The extracts were offered at a dosage of 50 mg/kg of feed DM intake based on the previous week’s feed intake record, and the dosage was adjusted weekly. This dosage was based on a previous dose–response in vitro study that established that 50 mg/kg substrate DM was effective in significantly reducing methane production [14]. 

The TMR was formulated to support an average daily gain (ADG) of 250 g/head/d. The lambs were adapted to the diet for an initial 14 d and a further 7 d gradual adaptation to the oral drenching with the respective extract solutions. After this 21 d adaptation period (mean BW, 35.6 ± 2.2), lambs were offered the respective dietary treatment for a continuous period of 76 d. The orts from the previous day’s feed were collected and weighed before the next feed was offered in order to determine each animal’s average DMI. Lambs were weighed at the start of the experiment and weekly thereafter. The average daily gain was calculated by plotting the weekly body weights against time in weeks, while feed conversion efficiency (FCE) was also calculated from DMI and weight gain.

### 2.2. Blood Collection and Analysis

At the end of the growth period, blood samples were taken from the jugular vein of all 40 lambs into two tubes, one containing ethylene diamine tetra acetic acid (EDTA) for hematological analysis and the second for serum chemical analysis. The samples were stored in a cooler box and transported immediately to the Veterinary Diagnostic Laboratory, University of Pretoria, Onderstepoort, South Africa, for analysis. Full blood count was conducted using a multi-parameter automated hematology analyzer (ADVIA 2120i, Siemens, Bavaria, Germany), and serum chemistry was analyzed using the Cobas Integra 400 Plus (Roche, Basel, Switzerland) for urea nitrogen, glucose, cholesterol, total serum protein, albumin, globulin, aspartate transaminase (AST), alanine transaminase (ALT), and alkaline phosphatase (ALP). The values obtained for each parameter were compared across treatments, while the outcome was discussed in relation to reference intervals for a 16-week-old Merino sheep [18].

### 2.3. Nutrient Digestibility and Nitrogen Balance Evaluation

After the growth study, one ram from each pen was randomly selected, and a total of five lambs per treatment were transferred into metabolic crates adapted for total fecal and urine collection. Adaptation to the crates was conducted for 5 d, during which lambs were adjusted to the fecal bags and crates. Samples of the feed, orts, feces, and urine were taken daily for 7 days and frozen at −20 °C for subsequent analysis. Urine was collected into funnel-shaped pans covered with a mesh cloth and placed under the metabolic crates. The urine collection bottles contained approximately 100 mL of 10% sulfuric acid (10% *v*/*v*; Merck Millipore KGaA, Darmstadt, Germany) to keep urine pH below 3. Thereafter, an aliquot of daily urine collection was sampled per animal over the 7 d period. At the end of the collection period, feed, orts, and feces for each animal were pooled across days and sub-sampled for analysis [15].

### 2.4. Methane Emission Measurement

At the end of the digestibility trial, one lamb representing each dietary treatment was placed inside the open-circuit respiratory chamber for the measurement of enteric methane emissions. All twenty lambs used for the digestibility trial were therefore used in five batches (four lambs per batch) to quantify methane emission over a 5-day period per batch. Each chamber was constructed with 25 × 25 mm powder-coated steel to create an area with an approximate volume of 5 m^3^. Recovery tests were carried out on all the chambers before each cycle of methane measurement by releasing a known volume of 99.5% methane from a pressurized cylinder into each chamber. Details of the chamber characteristics and recovery tests were as previously reported [15]. Recovery percentages obtained for the four chambers during the trial ranged from 78.2 to 92.9%. To minimize the effect of chamber differences as reflected by recovery rates, the lambs in each batch were rotated randomly across the 4 chambers over the 4 d collection period. Within each cycle of methane measurement, lambs were adjusted to the chamber for 1 d, and then methane data were collected over the remaining 4 days. All lambs were fed a fixed amount of the TMR and drenched with the extracts as described previously.

After methane emission measurement, lambs were fasted for 12 h before slaughtering, which involved electrical stunning and exsanguination. Within 30 min of slaughtering, the entire rumen content of each lamb was emptied into a plastic bucket, mixed, and sampled. The sampled rumen content was strained through 4 layers of cheesecloth into tubes containing 25% orthophosphoric acid for the analysis of volatile fatty acid. 

### 2.5. Chemical Analysis

Samples of feed, orts, and feces were weighed and oven-dried at 105 °C for 18 h to determine DM content [19], while a bulk sample was dried at 55 °C for 48 h, milled through a 1 mm screen, and stored for further chemical analysis. Milled samples of feed, feces, and orts were analyzed for organic matter and ash [20], while acid detergent fibre (ADF) and neutral detergent fibre (NDF) were analyzed using Ankom technology 200 (Ankom Technology, Fairport, NY, USA) [21]. The NDF assay was conducted with heat-stable alpha-amylase and sodium sulphite, and both NDF and ADF were expressed exclusively in residual ash. The nitrogen content of the feed, orts, feces, and urine was analyzed using the Leco Nitrogen/Protein analyzer (Leco TruMac N determinator Leco Corporation, Kirchheim, Germany). Volatile fatty acid analysis was carried out using a gas chromatography fitted with a Barrier Ionization Discharge (BID) detector (Shimadzu GC-2010 Tracera; Shimadzu Corp., Kyoto, Japan), and the protocol is as detailed by Adejoro et al. [15].

### 2.6. Statistical Analysis

Feed consumption during the growth trial was averaged for two lambs within each pen, and together with the body weight for each animal was used to calculate FCR. Individual animal data during the growth, digestibility, and methane emission measurement were grouped according to treatment and analyzed using the Proc mixed procedures of SAS 9.4 (SAS Institute Inc., 2004. Cary, NC, USA), and the model statement includes

(1) $$Yij=µ+\alpha_{i}+\beta_{j}+{(\alpha \beta }_{ij})+\varepsilon_{ij}$$

where Yij is the response of different treatments; µ is the overall mean; $\alpha_{i}$, treatment effect; $\beta_{j}$ is blocking effect; ${\alpha \beta }_{ij}$ is the interaction effect of treatment and blocks; and $\varepsilon_{ij}$ is the effect of random error. The block effect, as well as the interaction effect of treatment and block, were random factors, while the treatment effect (diet) was a fixed effect. 

For methane emission, the model statement includes

$$Yij=µ+\beta_{i}+\alpha_{j}+\varepsilon_{ij}$$

where Yij is the response of different treatments; µ is the overall mean; $\beta_{i}$ is the effect of incubation runs; $\alpha_{j}$, treatment effect; and $\varepsilon_{ij}$ is the effect of random error. The effect of incubation runs was a random factor, while the treatment effect (diet) was a fixed effect.

Significant means were separated using the Tukey test and significance was established when *p* ≤ 0.05 and a trend at 0.05 < *p* ≤ 0.10 and non-significance when *p* > 0.10.

## 3. Results

### 3.1. Animal Performance and Nutrient Digestibility

There were no differences in nutrient intake and apparent digestibility of DM, OM, NDF, ADF, and CP in the lambs receiving Monensin, Neem, or Moringa leaf extract (*p* > 0.05) (Table 2). Equally, fecal-N, urine-N, and overall nitrogen retention were not different across dietary treatments (*p* > 0.05). The ADG of lambs was not affected by dietary treatments (*p* > 0.05), and lambs gained between 295 and 314 g/d during the growth phase (Table 3). Equally, the feed conversion ratio was not affected by the intake of the extracts or Monensin. Additives only showed a tendency for reduced methane production when expressed in g/day (*p* = 0.08) and g/kg DM intake (*p* = 0.07). 

The extracts did not have any effect on total volatile fatty acid (TVFA) production, as well as on butyric and valeric acid proportions in the rumen (Table 4). However, extracts resulted in significant differences in acetic acid (*p* < 0.02) and propionic acid (*p* < 0.03) proportion in the rumen, with animals receiving Neem having significantly higher acetic acid and lower propionic acid compared to animals on the control, Monensin or Moringa extract. The branched-chain VFA proportion was least in the control, followed by Neem, and then Moringa, and was highest in Monensin-supplemented animals. 

### 3.2. Hematological and Serum Biochemical Profile

Hemoglobin, red blood cell count (RBC), white blood cell count (WBC), hematocrit value, mean corpuscular volume (MCV), mean corpuscular hemoglobin (MCH), mean corpuscular hemoglobin concentration (MCHC), segmented neutrophil, lymphocytes, monocytes, eosinophil, and basophil counts were all within the reference range [18] and did not differ (*p* > 0.05) among the treatments (Table 5). In contrast, lambs receiving Monensin and Neem extract had higher (*p* < 0.05) platelet counts compared to lambs in the control group. Urea-nitrogen, glucose, cholesterol, total serum protein, and albumin concentration in the blood were not affected by plant extracts supplementation (*p* > 0.05) (Table 6). However, globulin concentration was different across the treatments (*p* < 0.05), with Monensin-supplemented lambs having higher blood globulin compared to the control and Neem-treated animals. Nevertheless, liver enzymes were not affected by plant extract supplementation as the aspartate aminotransferase (AST), alanine aminotransferase (ALT), and alkaline phosphatase (ALP) concentrations were not different across the treatments (*p* > 0.05). The liver enzyme concentrations were also within the normal range ascribed to growing lambs.

## 4. Discussion

### Digestibility, N-Balance, and Growth Performance

*Moringa oleifera* (Moringa) and *Azadirachta indica* (Neem) are multipurpose trees which have continued to attract wide attention from scientists and farmers. They provide shade, their foliage is rich in protein, and many are drought tolerant, hence well suited for the various livestock production systems across the tropics and subtropics [22]. Therefore, a potential methane mitigation attribute of their extracts would only be an added benefit to the already established value among farmers. Only a few studies have evaluated the rumen-modulating effects of Neem and Moringa extracts in live animals, although several in vitro studies have reported their potential to improve substrate fermentation and reduce enteric methane production [23,24,25], which therefore limits their application in commercial livestock production [14,26].

While it was hypothesized that plants extracts can improve growth performance in ruminants via increased energy utilization, ammonia recycling, or improved fibre digestion [14,27], responses in vivo have been very variable, and this variability can be associated with the type, level of extract and nature of the secondary metabolites present in the plant extracts [27]. Following prolonged feeding, Neem and Moringa extract at 50 mg/kg DM did not show any effect on nutrient digestibility and lamb growth. In this study, there were no changes in nutrient intake and digestibility, although authors reported [16] significant improvement in nutrient intake and dry matter digestibility when sheep were orally drenched with 40 mL/day of Moringa extract. Retained nitrogen was channelled into various biological pathways such as muscle and wool growth, all of which are not 100% efficient, thus accounting for the reported ADG being less than the estimated ADG based on N-retention. 

While Moringa leaves contain appreciable amounts of tannins (mainly hydrolysable tannin), saponin, and phytate [28], Neem foliage is known to contain high contents of condensed tannin, saponins, oxalate, and limonoids such as azadirachtin [6,26,29]. At moderate to high concentrations, these metabolites are expected to exert a significant effect on rumen fermentation, such as modulating microbial enzymes, bacteria attachment to feed, or N-recycling [22,30]. Controlling microbial degradation of dietary CP in the rumen could effectively reduce N losses and improve the efficiency of N utilization [15,31]. Both Monensin and Moringa did not affect TVFA or shift VFA patterns, and this is similar to the observed responses in a Rusitec system [32].

Monensin, Moringa, and Neem reduced methane (CH_4_ per kg DMI) by 20%, 24%, and 15%, respectively, although the decrease only showed a tendency for significance. This may be due to wide variability within the same treatment across the methane emission runs. This result downplayed previous in vitro studies, which projected Moringa and Neem extract as potent anti-methanogenic additives [14,32]. A previous study [16] estimated methane production based on the volatile fatty acid concentration of rumen fluid in sheep, so the results may not be rigorously compared with the current study. Saponin, which is present in Moringa, is a known antiprotozoal agent, but authors [30] noted that only 10% to 20% of methanogens are associated with protozoa; thus, the sole inhibition of protozoal activity may not result in a significant reduction in methane emission. Furthermore, it appears that the dose used in the current study, although effective in vitro [9], may only produce marginal anti-methanogenic effects in vivo or that the rumen microbiome developed adaptation to the PSMs, as noted by previous studies [33]. 

Apart from the direct antimicrobial effects of PSM in the rumen and post-rumen, PSMs are known to bind specific receptors expressed in neurons, intestines, and other cells and exhibit physiological effects similar to those observed in non-ruminants [34]. For example, a high concentration of azadirachtin, the main alkaloid in Neem extract, was detectable in blood plasma, suggesting that Neem extract was resilient to chemical modification by ruminal microbial action before reaching peripheral circulation [35]. This therefore opens wide research opportunities to explore their economic benefits in ruminant animals, such as hepatoprotective and neuroprotective effects on the animal, among others. Equally important is the potential cytotoxic effect of PSMs when animals consume them in high-enough doses. 

Although the current result showed that extracts did not have any effect on most hematology indices, such as the WBC and lymphocytes, which partake in immune system regulation, previous studies have shown that high doses of Neem extract could enhance the immune cells, as observed with reduced pro-tumour inflammatory cytokines in rat models, confirming the health status-enhancing benefits of Neem extract [36]. Like the current study, lymphocyte count in goats was not affected by Moringa leaf meal supplementation, although the goats showed higher WBC compared to the controlled animals [37]. The concentration and bioactivity of PSMs generally determine their antioxidant or immune cell-enhancing properties [34]. Platelet counts across the treatment groups were above the level that signals the onset of clinical thrombocytopenia [38].

Ammonia is a product of protein degradation by rumen microbes, which they utilize in the synthesis of their microbial protein [39]. Excess ammonia is absorbed into the portal circulation, where it is converted to urea by hepatocytes in the liver and subsequently either excreted in the urine or recycled back to the gut [40]. The lack of significance in blood urea nitrogen (BUN) is consistent with the rumen ammonia and urine nitrogen excretion trends observed in the current study. The concentration of BUN can give an indication of liver function [41]. The more accurate biomarkers to determine hepatoxicity are the enzymes alanine aminotransferase (ALT), which is a liver-specific enzyme, or aspartate aminotransferase (AST) and alkaline phosphatase (ALP), which are indicators of liver or tissue damage or indicate release of inflammatory cytokines [17,42]. The lack of changes in ALT, AST, and ALP values across the treatments confirms the earlier report [35], which observed that a 6 g/kg Neem extract diet for the control of ticks did not show any significant change in ALT, AST, and ALP concentration. Furthermore, Raizada et al. [43] reported that the LD50 dose for azadirachtin, the main alkaloid in Neem, was above 1500 mg/kg BW in rats. The doses used by Raizada et al. [43], as well as by Landau et al. [35], were more than ten-fold higher than the dose of Neem extract used in the current study. Similarly, Moringa extract doses up to 600 mg/kg BW did not cause any negative change in liver enzymes in rat models [23].

## 5. Conclusions

Results from this study showed that prolonged oral drenching of 50 mg/kg DMI with crude Neem or Moringa leaf extract did not affect the performance or blood profile of the growing lambs. The extracts also showed a tendency for reduced methane, which should warrant further investigation to validate their anti-methanogenic credentials.

## Figures and Tables

**Table 1:** Ingredient (% as is) and chemical composition of the total mixed ration fed to experimental animals for the duration of the experimental trial.

Ingredient	Composition (%)
Soybean meal	17
Maize (corn) grain	28
Lucerne hay	20
Eragrostis hay	22.7
Sugarcane Molasses	6
Wheat bran	5
Urea	0.8
* Vitamin–mineral premix	0.5
Total	100
Analyzed chemical composition	
Dry matter (%)	89.6
Crude protein (g/kg DM)	192
Starch (g/kg DM)	221
Neutral detergent fibre (g/kg DM)	324
Acid detergent fibre (g/kg DM)	186
Acid detergent lignin (g/kg DM)	32
Crude ash (g/kg DM)	71.4
Metabolizable energy (MJ/kg DM)	10.6

* Premix contains the following (g/kg): vit A, 18,000 IU; vit D, 3920 IU; vit E, 2.45 IU; Zn, 5.0 mg; Mn, 4.1 mg; Cu, 0.5 mg; Se, 0.2 mg; Mg, 28 mg; and Co, 0.3 mg.

**Table 2:** Effects of Monensin, or methanolic extracts of Moringa or Neem on the nutrient intake, digestibility, and nitrogen excretion in South African Mutton Merino lambs.

Parameters	Control	Monensin	Moringa	Neem	SEM	*p*-Value
Nutrient intake (g/d)						
Dry matter	1672	1866	1765	1655	37.0	0.25
Neutral detergent fibre	587	650	635	586	15.5	0.39
Acid detergent fibre	277	328	310	286	9.83	0.24
Crude protein	346	402	362	339	9.44	0.11
Apparent Nutrient Digestibility (g/100 g DM)						
Dry matter	68.9	68.4	68.2	65.3	0.61	0.35
Organic matter	68.7	67.9	68.0	64.6	0.65	0.29
Neutral detergent fibre	49.0	48.9	50.5	41.8	1.42	0.16
Acid detergent fibre	37.1	40.1	43.2	34.1	1.75	0.65
Crude protein	79.2	78.2	78.0	78.5	0.41	0.61
Fecal-N (g/d)	10.3	10.5	11.6	12.3	0.40	0.34
Urinary-N (g/d)	17.5	13.2	19.2	17.5	0.54	0.38
N-retained, g/d	21.6	24.5	22.5	27.4	0.83	0.38

Monensin sodium as a positive control (5 mg/kg DM); Moringa and Neem extracts offered at 50 mg/kg DM feed.

**Table 3:** Growth performance and methane emission of South Africa Mutton Merino lambs as influenced by dietary Monensin or methanolic extracts of Moringa or Neem leaf.

Parameters	Control	Monensin	Moringa	Neem	SEM	*p*-Value
Initial BW (kg)	35.6	35.5	35.6	36.0	1.32	0.99
Final BW (kg)	59.0	59.0	60.0	59.2	1.26	0.99
Average daily gain (g/d)	295	309	314	302	5.95	0.73
Average dry matter intake (g/d)	2051	1941	1806	1781	125	0.10
Feed conversion ratio	7.4	7.2	6.9	7.2	0.16	0.79
CH_4_ emitted (g/d)	36.9	31.6	30.3	33.5	0.99	0.08
CH_4_ emitted (g/kg DMI)	22.2	17.7	16.8	18.9	0.73	0.07

**Table 4:** Rumen volatile fatty acid concentration in South Africa Mutton Merino lambs receiving dietary Monensin, extracts of Moringa (*Moringa oleifera*), or Neem (*Azadirachta indica*) leaf.

Parameters	Control	Monensin	Moringa	Neem	SEM	*p*-Value
Total volatile fatty acid (mmol/L)	151	137	144	142	2.60	0.39
Acetic acid (mol/100 mol)	59.9 ^b^	61.3 ^b^	60.8 ^b^	62.7 ^a^	0.37	0.02
Propionic acid (mol/100 mol)	22.7 ^a^	21.8 ^a^	22.2 ^a^	19.6 ^b^	0.43	0.03
Butyric acid (mol/100 mol)	12.6	12.8	11.4	12.5	0.23	0.09
Valeric acid (mol/100 mol)	1.41	1.40	1.43	1.34	0.03	0.18
Branched VFA (mol/100 mol)	3.41 ^c^	4.83 ^a^	4.16 ^b^	3.77 ^b^	0.30	0.04
Acetic acid: Propionic acid ratio	2.64 ^b^	2.81 ^b^	2.70 ^b^	3.34 ^a^	0.14	0.04

Branched-chain VFA = Isobutyric acid + Isovaleric acid. Means across the same row with different letters are significantly different (*p* < 0.05).

**Table 5:** Hematological parameters of South Africa Mutton Merino lambs consuming a total mixed ration with Monensin, Moringa (*Moringa oleifera*), or Neem (*Azadirachta indica*) leaf extract.

^1^ Parameters	^2^ Reference Range	Control	Monensin	Moringa	Neem	SEM	*p*-Values
Hemoglobin (g/L)	105–137	117	111	115	111	1.71	0.46
RBC (×10^12^/L)	9.2–13.0	10.8	10.6	10.7	10.2	0.18	0.52
WBC (×10^9^/L)	5.1–15.9	6.2	7.0	6.9	6.9	0.27	0.74
Hematocrit value (L/L)	0.28–0.39	0.3	0.4	0.4	0.4	0.01	0.38
MCV (fL)	28–35	35.2	33.9	34.6	34.8	0.34	0.51
MCH (pg)	10–13	10.9	10.5	10.7	10.9	0.10	0.48
MCHC (g/dL)	31.7–39.2	31.0	31.1	31.5	31.2	0.12	0.47
Red cell distribution (%)	NFR	16.7	17.4	16.9	17.0	0.20	0.52
Segmented Neutrophil (×10^9^/L)	0.8–6.3	2.2	2.9	2.7	2.7	0.17	0.53
Lymphocytes (×10^9^/L)	2.1–10.2	3.8	3.7	4.0	3.9	0.13	0.93
Monocyte (×10^9^/L)	0.1–0.8	0.2	0.3	0.3	0.2	0.03	0.36
Eosinophil (×10^9^/L)	0–0.2	0.09	0.05	0.04	0.1	0.01	0.11
Basophil (×10^9^/L)	0–0.2	0.00	0.04	0.02	0.01	0.01	0.14
Platelet count (×10^9^/L)	426–1142	313 ^b^	552 ^a^	480 ^ab^	529 ^a^	32.7	0.02

^1^ RBC, red blood cell count; WBC, white blood cell count; MCV, mean corpuscular volume; MCH, Mean corpuscular hemoglobin; MCHC, Mean cell hemoglobin concentration. ^2^ Reference intervals as determined [18]. SEM: standard error mean. Means across the same row with different letters are significantly different (*p* < 0.05).

**Table 6:** Serum chemistry parameters in South Africa Mutton Merino lambs receiving a total mixed ration with Monensin or extracts of Moringa (*Moringa oleifera*) or Neem (*Azadirachta indica*) leaf.

^1^ Parameters	^2^ Reference Range	Control	Monensin	Moringa	Neem	SEM	*p*-Values
Urea N (mmol/L)	5.0–9.1	11.6	13.1	11.6	12.3	0.25	0.13
Glucose (mmol/L)	2.7–4.8	4.2	4.3	4.3	4.3	0.04	0.91
Cholesterol (mmol/L)	NFR	1.5	1.4	1.5	1.5	0.04	0.23
TSP (g/L)	51–64	62.2	65.2	62.9	62.5	0.53	0.10
Albumin (g/L)	30–37	39.5	39.7	39.3	39.6	0.29	0.95
Globulin (g/L)	19–30	22.8 ^b^	25.6 ^a^	23.6 ^ab^	23 ^b^	0.44	0.03
AST (U/L)	83–140	111	120	110	111	3.89	0.80
ALT (U/L)	9–45	13.6	13.8	16.3	13.9	0.64	0.28
ALP (U/L)	99–464	373	380	298	364	18.6	0.35

^1^ TSP, total serum protein; AST, Aspartate aminotransferase; ALT, Alanine aminotransferase, ALP, Alkaline phosphatase. ^2^ Reference intervals as reported [18]. SEM, standard error mean. NFR: No reference range. Means across the same row with different letters are significantly different (*p* < 0.05).

## Data Availability

The authors confirm that the data supporting the findings of this study are available within the article.
